# Neonatal airway emergency: congenital laryngeal myxoma

**DOI:** 10.1093/jscr/rjag904

**Published:** 2026-10-10

**Authors:** Haifa K Alqahtani, Hesham S Almofada, Mohammed Dababo, Fahad Z Alotaibi

**Affiliations:** College of Medicine, King Saud University, PO Box 2925, Riyadh 11461, Saudi Arabia; Department of Otolaryngology–Head and Neck Surgery, King Faisal Specialist Hospital and Research Centre (KFSHRC), PO Box 3354, Riyadh 11211, Saudi Arabia; Department of Pathology and Laboratory Medicine (MBC 10), King Faisal Specialist Hospital and Research Centre (KFSHRC), PO Box 3354, Riyadh 11211, Saudi Arabia; Department of Otorhinolaryngology–Head and Neck Surgery, Imam Mohammad Ibn Saud Islamic University, Othman Bin Affan Road, Alnada-7544, Riyadh 13317-4233, Saudi Arabia

**Keywords:** laryngeal myxoma, congenital, neonate, stridor, vocal cord, post-intubation granuloma

## Abstract

Laryngeal myxomas are exceptionally rare benign mesenchymal tumors, particularly in the pediatric population. We present a case of a neonate with a history of brief endotracheal intubation who developed inspiratory stridor and respiratory distress. Flexible laryngoscopy revealed a pedunculated mass on the right true vocal cord. The lesion was completely excised via microlaryngobronchoscopy. Definitive histopathological and immunohistochemical analysis—showing a hypocellular myxoid stroma, bland spindle cells, a low Ki-67 proliferation index (<5%), and positivity for CD34 with negative staining for S-100, desmin, smooth muscle actin, myogenin, ALK1, and **β**-catenin—confirmed the diagnosis of a congenital laryngeal myxoma, differentiating it from a reactive post-intubation granuloma. This case highlights the importance of definitive histopathological diagnosis over presumptive clinical history and emphasizes the inclusion of rare congenital neoplasms in the differential diagnosis of neonatal stridor. Complete surgical excision is the treatment of choice.

## Introduction

Neonatal stridor is a critical sign indicating upper airway obstruction that necessitates prompt endoscopic evaluation. While laryngomalacia is the most common etiology [1], a broad differential diagnosis must be maintained. This includes congenital cysts, hemangiomas, and true neoplasms [2]. Crucially, only endoscopy provides a definitive diagnosis, as the clinical presentation of various laryngeal lesions can be indistinguishable.

Laryngeal myxomas are benign tumors of mesenchymal origin. In the head and neck region, myxomas most commonly arise in the mandible and maxilla. The laryngeal localization is exceedingly rare. A review of the published literature reveals approximately 30 reported cases, with a strong predilection for middle-aged adult males and a history of smoking [3–6]. The occurrence of a laryngeal myxoma in a neonate is, to our knowledge, an exceptional finding.

The diagnosis of a laryngeal myxoma in a neonate is complicated when a history of airway instrumentation suggests a more common reactive lesion, such as a post-intubation granuloma. We present this case to illustrate that definitive histopathological evidence must take precedence over a potentially misleading clinical history.

## Case report

A male neonate was delivered via cesarean section at 37 + 2 weeks’ gestation with intrauterine growth restriction. He developed immediate respiratory distress, requiring endotracheal intubation on Day 1 of life. He was extubated on Day 5, re-intubated for a separate procedure on Day 7, and extubated again on Day 8. The total duration of intubation was approximately 6 days.

On Day 9 of life, the infant presented with acute onset inspiratory stridor, intermittent oxygen desaturation, and a weak cry. Flexible laryngoscopy revealed a pedunculated, smooth, reddish lesion on the middle third of the right true vocal cord, with bilaterally mobile vocal cords (Fig. 1). Given the recent intubation history, a post-intubation granuloma was initially considered; however, the rapid onset of symptoms prompted consideration of a pre-existing congenital mass.

**Figure 1:**
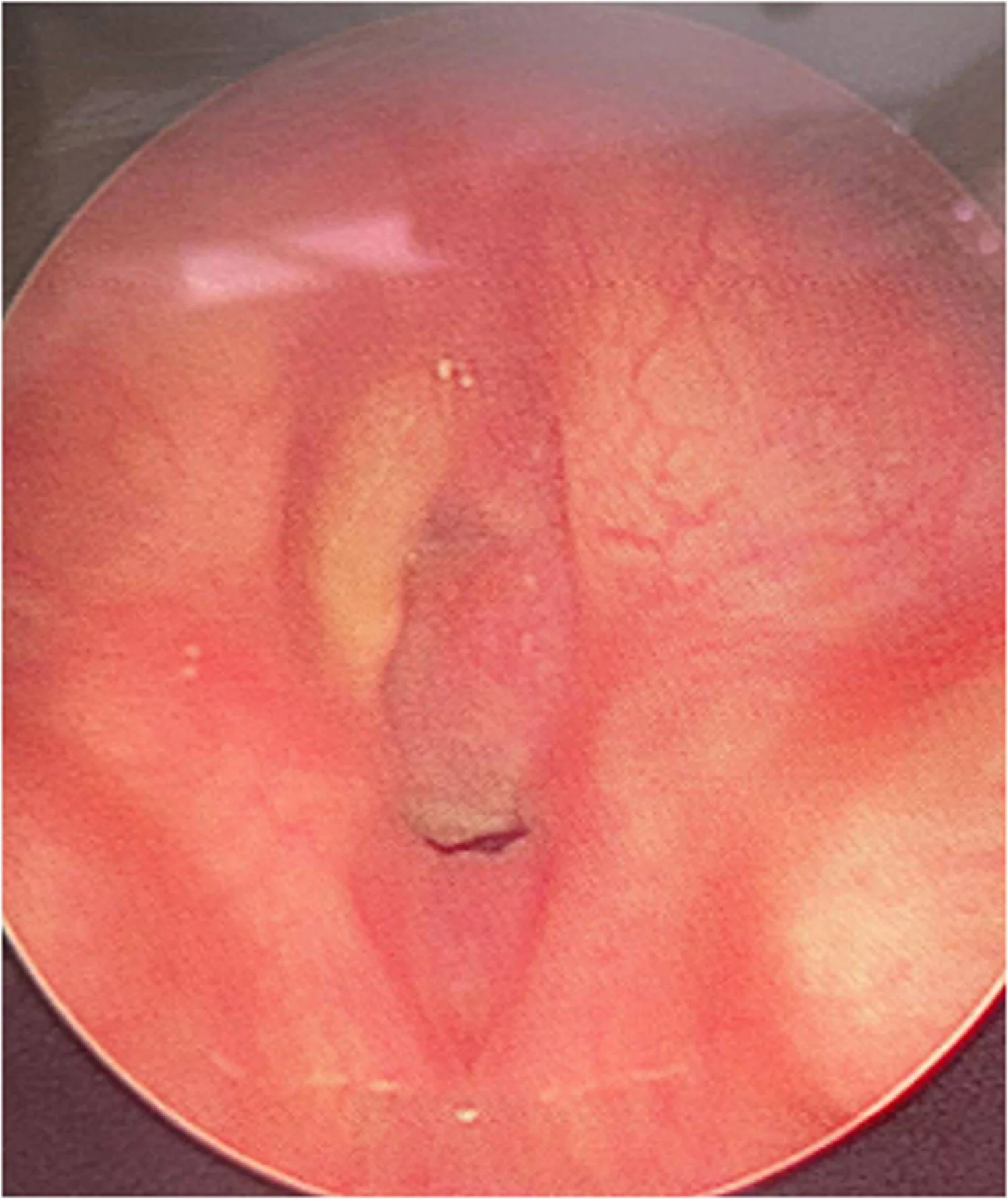
Direct laryngoscopy view of the right vocal cord lesion on Day 9 of life. A pedunculated, smooth, reddish lesion is visible on the middle third of the right true vocal cord. The vocal cords were noted to be bilaterally mobile.

On Day 10 of life, the patient underwent microlaryngobronchoscopy under general anesthesia, and the mass was completely excised using cold steel instrumentation. The postoperative course was uneventful, and he was discharged home 48 h later.

Histopathological examination of the excised specimen revealed a subepithelial lesion composed of a hypocellular myxoid stroma containing scattered spindle and stellate cells with bland nuclei, without pleomorphism, mitotic activity, or necrosis (Fig. 2). The lesional spindle cells were positive for CD34 and negative for S-100, desmin, myogenin, smooth muscle actin, ALK1, and β-catenin. The Ki-67 proliferation index was very low (<5%). These definitive histopathological and immunohistochemical findings are diagnostic of a laryngeal myxoma, excluding a reactive granuloma and other mesenchymal neoplasms.

**Figure 2:**
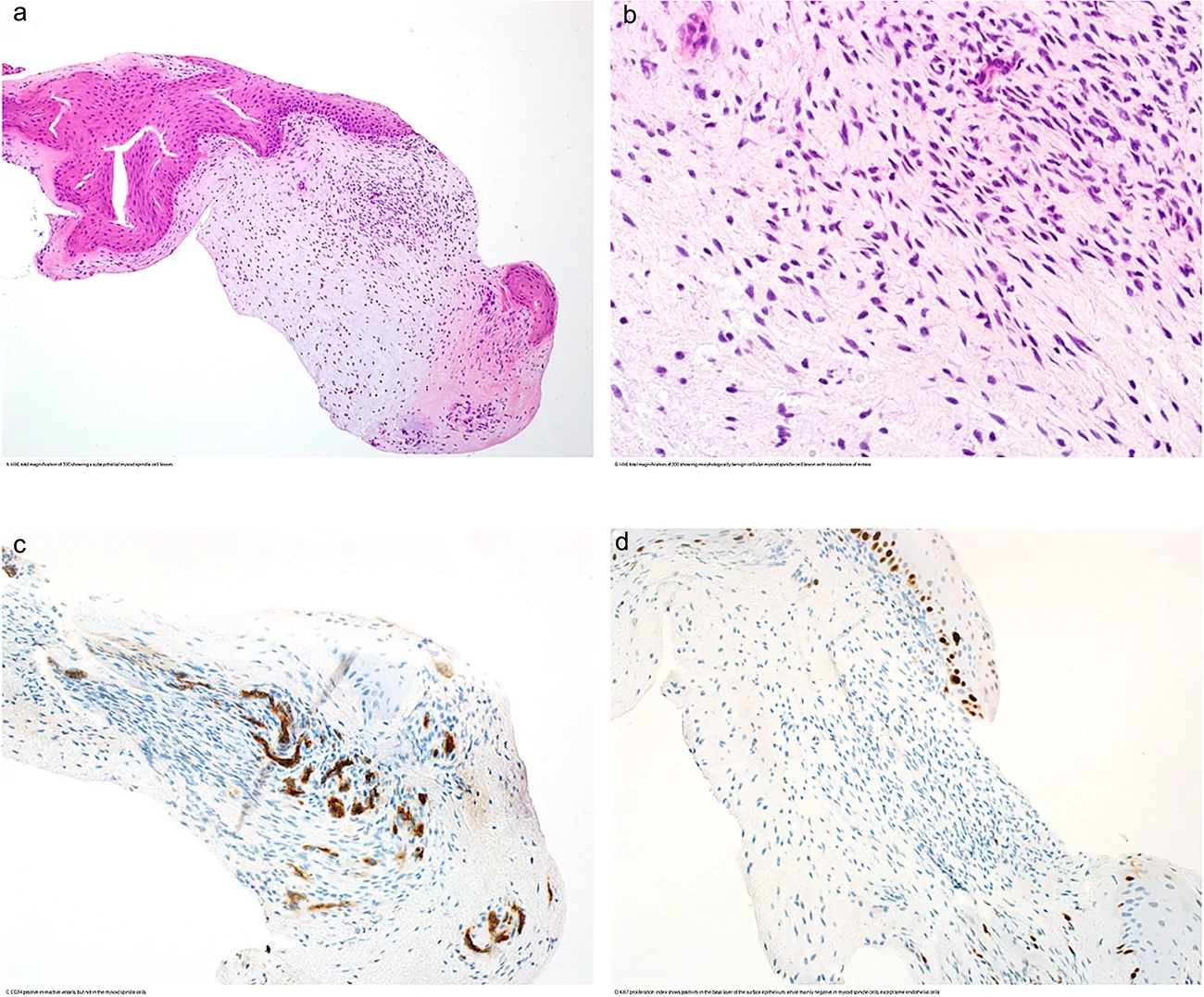
Histopathological and immunohistochemical findings. (a) Hematoxylin & Eosin (H&E) stain (100× magnification) showing a subepithelial myxoid spindle cell lesion. (b) H&E stain (200× magnification) showing a morphologically benign cellular myxoid spindle cell lesion with no evidence of mitosis or nuclear atypia. (c) CD34 immunohistochemistry, showing positivity in reactive vessels. (d) Ki-67 proliferation index, showing positivity in the basal layer of the surface epithelium but largely negative (<5%) in the myxoid spindle cells, confirming the low proliferative activity of the lesion.

At the follow-up appointment 3 months post-surgery, the patient remained asymptomatic. Laryngoscopy confirmed a well-healed vocal cord with no evidence of recurrence (Fig. 3).

**Figure 3:**
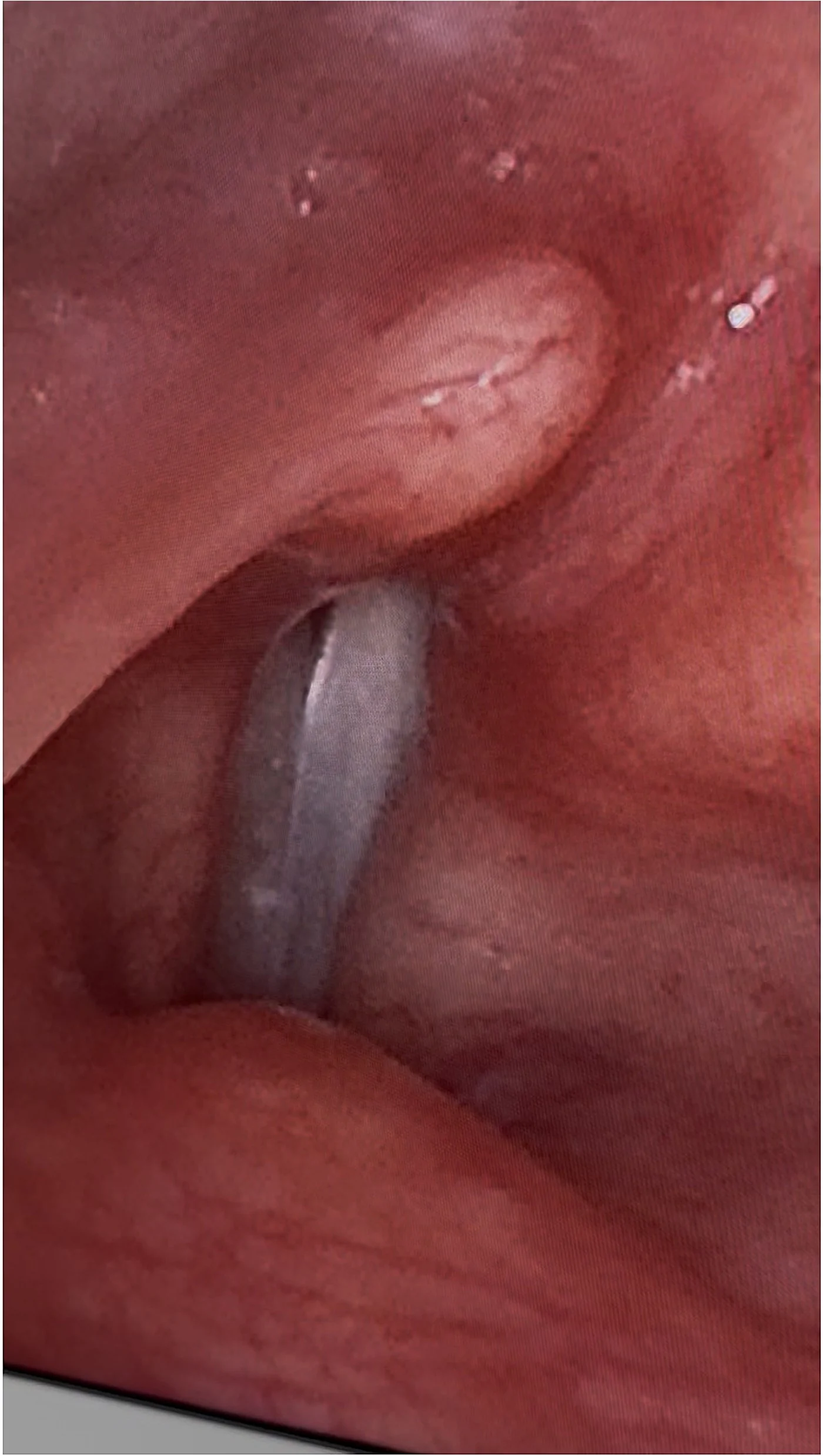
Flexible laryngoscopy view at the 3-month outpatient follow-up. The right vocal cord is well-healed with no evidence of scarring or recurrence of the lesion, confirming complete excision.

## Discussion

This case presents a unique diagnostic challenge: a neonatal vocal cord mass in the context of a recent intubation history. The argument against a post-intubation granuloma is twofold. First, the clinical timeline is inconsistent. Granulomas require a period of mucosal injury and organized tissue repair to form a distinct mass. The lesion was identified just 1 day after extubation, making it far more plausible that it was a pre-existing congenital lesion that became clinically apparent due to mucosal edema secondary to intubation. Second, the histopathology was incompatible with a reactive granuloma. A typical intubation granuloma is characterized by dense inflammatory infiltrate and robust capillary proliferation. In contrast, the excised lesion was defined by a prominent hypocellular myxoid stroma and bland spindle cell morphology [5, 6].

Laryngeal myxoma is an exceptionally rare entity. The systematic review by Yu *et al*. (2021) identified 24 cases with a mean patient age of 53.3 years and a strong male predominance [4]. Recent publications by Mnejja *et al*. and Bilgin *et al*. report cases with slow and progressive onset of hoarseness over months to years [7, 8]. The onset of stridor in our neonate was acute, occurring within days of birth, consistent with a congenital lesion rapidly unmasked by the physiological stress of extubation.

The immunohistochemical profile is central to the definitive diagnosis of myxoma. The differential diagnosis of a myxoid spindle cell lesion includes myxoid neurofibroma (S-100 positive), low-grade myxofibrosarcoma, low-grade fibromyxoid sarcoma, and rhabdomyosarcoma (desmin and myogenin positive) [6]. In our case, the comprehensive negative immunoprofile for all these markers, combined with the characteristic histological appearance, conclusively established the diagnosis of myxoma. The CD34 positivity observed supports the diagnosis, though it is not universally present [3, 8].

Complete surgical excision via microlaryngoscopy using cold instruments is the gold standard of treatment [7, 9]. Although myxomas are benign, they lack a true fibrous capsule and can infiltrate surrounding tissues, carrying a risk of recurrence if incompletely resected [3, 8]. Recurrence in the laryngeal site appears to be less common than for myxomas elsewhere in the head and neck, but long-term endoscopic follow-up is necessary [7].

## Data Availability

All data gathered during this research are available from the corresponding author upon reasonable request.
